# Nervous and Mental Diseases

**Published:** 1899-08

**Authors:** 


NERVOUS AND MENTAL DISEASES. By ARCHIBALD CHURCH, M. D., Professor of Clinical Neurology and Mental Diseases in the Northwestern University Medical School, Chicago; Professor of Neurology in Chicago Polyclinic and Neurologist to St. Luke's Hospital, Chicago, etc., etc.; and FREDERICK PETERSON, M. D., Clinical Professor of Mental Diseases in Woman's Medical College, New York, and Chief of Clinic, Nervous Department, College of Physicians and Surgeons, New York. Pp. 842; 800, with 305 illustrations. Philadelphia: W. B. Saunders, Publisher. 1899.

The author says of their work that it "is not the joint work of two writers, but each author—Dr. Church in Neurology and Dr. Peterson in Psychiatry—has contributed to the making of a single volume; each is, therefore, solely responsible for the work in his own department."

A few years ago there existed but one text-book on nervous diseases by an American author; now we have them almost by the dozen, and all creditable to their authors and to American neurology. The new comer finds itself in most excellent company and all things considered has very little to feel ashamed about. Doctor Church has certainly given us a very fine and valuable addition to American text-books and has treated his subject with great care and good judgment, not only in regard to facts warranted, but to the opinions of his co-workers. Dr. Peterson has restricted his part on pyschiatry to his own work largely. and the great bulk of his references are to his own published articles in the various journals.

This perhaps is not wise in a book written for medical students and general practitioners as it gives the impression of exclusiveness—which although a desideratum in monographs—is yet not so desirable in a general text-book. To those more acquainted with Dr. Peterson's excellent original work, this book serves practically as a "gesammelte Arbeiten" of the Germans.

Dr. Church divides nervous diseases into eight parts, and these into chapters, dealing with the different forms of nerve, cord and brain disease, diseases of the nervous system, without known anatomical basis, symptomatic disorders, and examination of patients.

Under the neuroses or those affections without known pathological lesion, Church includes acromegaly, myxedema, exophthalmic goitre, tetanus, hysteria, neurasthenia and other so-called neuroses. He therefore is not a staunch believer in the theory that the thyroid, thymus, pituitary and other glands are solely responsible for these obscure affections, but is willing to wait for more conclusive pathological testimony. In speaking of hysteria, Dr. Church sides with the French school and gives Charcot and his school full credit for placing this malady on a firm clinical basis.

Dr. Peterson divides his part into thirteen chapters, as follows: Chapter I., on definition and classifications of insanity; Chapter II., on general etiology; Chapter III., on symptomatology; Chapter IV., on examination of the patient; Chapter V., on general treatment, and Chapter VI. to XIII., on mania, melancholia, circular insanity, epileptic insanity, dementia, paralytic dementia, paranoia and idiocy respectively. These chapters are all written in Dr. Peterson's vigorous style and give a very careful and lucid description of these different mental states.

The book as a whole is a very commendable one, profusely illustrated, carefully written and represents to a very large degree the present status of neurology.

The publishers have aided materially in making it a handsome book in every respect. W. C. K.

